# 3-Ammonio­pyridinium tetra­bromido­mercurate(II) monohydrate

**DOI:** 10.1107/S1600536808012336

**Published:** 2008-05-03

**Authors:** Basem Fares Ali, Rawhi H. Al-Far, Salim F. Haddad

**Affiliations:** a Department of Chemistry, Al al-Bayt University, Mafraq 25113, Jordan; b Faculty of Information Technology and Science, Al-Balqa’a Applied University, Salt, Jordan; c Department of Chemistry, The University of Jordan, Amman, Jordan

## Abstract

The asymmetric unit of the title compound, (C_5_H_8_N_2_)[HgBr_4_]·H_2_O, consists of one cation, one anion and one water mol­ecule. The anion exhibits a distorted tetra­hedral arrangement about the Hg atom. The crystal structure contains alternating sheets of cations (in the *ac* plane) and stacks of anions. Several strong hydrogen-bonding inter­actions (pyN—H⋯Br and C—H⋯Br; py is pyridine), along with O—H⋯Br inter­actions, connect the sheets of cations to the stacks of anions. Cation–cation π–π stacking is also present (C⋯C distances in the range 3.424–3.865 Å). The shortest Br⋯Br distance is 3.9527 (9) Å.

## Related literature

For general background, see: Al-Far & Ali (2007*a* ▶); Desiraju (1997 ▶). For related literature, see: Al-Far, Ali & Al-Sou’od (2006 ▶); Al-Far & Ali (2007*b* ▶); Ali & Al-Far (2007*a* ▶, 2007*b* ▶, 2008 ▶); Ali, Al-Far & Haddad (2008 ▶). For bond distances see: Orpen *et al.* (1989 ▶).
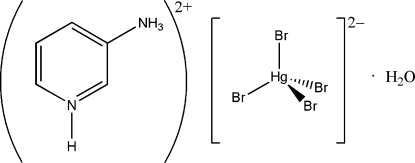


## Experimental

### 

#### Crystal data

(C_5_H_8_N_2_)[HgBr_4_]·H_2_O
*M* *_r_* = 634.34
Monoclinic, 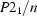

*a* = 8.1896 (7) Å
*b* = 14.0245 (12) Å
*c* = 11.5711 (10) Å
β = 94.730 (2)°
*V* = 1324.5 (2) Å^3^
*Z* = 4
Mo *K*α radiation
μ = 23.66 mm^−1^
*T* = 296 (2) K
0.20 × 0.10 × 0.03 mm

#### Data collection

Bruker–Siemens SMART APEX diffractometer
Absorption correction: multi-scan (*SADABS*; Bruker, 2001 ▶) *T*_min_ = 0.034, *T*_max_ = 0.492
16873 measured reflections
3780 independent reflections
2628 reflections with *I* > 2σ(*I*)
*R*_int_ = 0.056

#### Refinement

*R*[*F* ^2^ > 2σ(*F* ^2^)] = 0.035
*wR*(*F* ^2^) = 0.074
*S* = 1.03
3780 reflections
126 parameters
3 restraints
H atoms treated by a mixture of independent and constrained refinement
Δρ_max_ = 0.85 e Å^−3^
Δρ_min_ = −1.32 e Å^−3^

### 

Data collection: *SMART* (Bruker, 2001 ▶); cell refinement: *SAINT-Plus* (Bruker, 2001 ▶); data reduction: *SAINT-Plus*; program(s) used to solve structure: *XS* in *SHELXTL* (Sheldrick, 2008 ▶); program(s) used to refine structure: *XL* in *SHELXTL*; molecular graphics: *XP* in *SHELXTL*; software used to prepare material for publication: *XCIF* in *SHELXTL*.

## Supplementary Material

Crystal structure: contains datablocks I, global. DOI: 10.1107/S1600536808012336/cs2074sup1.cif

Structure factors: contains datablocks I. DOI: 10.1107/S1600536808012336/cs2074Isup2.hkl

Additional supplementary materials:  crystallographic information; 3D view; checkCIF report

## Figures and Tables

**Table:** 

Hg1—Br4	2.5818 (7)
Hg1—Br1	2.5875 (6)
Hg1—Br3	2.6216 (7)
Hg1—Br2	2.6309 (7)

**Table:** 

Br4—Hg1—Br1	120.99 (2)
Br4—Hg1—Br3	104.23 (2)
Br1—Hg1—Br3	109.78 (2)
Br4—Hg1—Br2	103.42 (2)
Br1—Hg1—Br2	107.40 (2)
Br3—Hg1—Br2	110.73 (2)

**Table 2:** Hydrogen-bond geometry (Å, °)

*D*—H⋯*A*	*D*—H	H⋯*A*	*D*⋯*A*	*D*—H⋯*A*
O1—H1⋯Br2^i^	0.89 (6)	2.98 (6)	3.564 (5)	125 (5)
O1—H2⋯Br3	0.89 (6)	2.65 (3)	3.513 (5)	162 (7)
N1—H1*A*⋯O1^ii^	0.89	1.80	2.686 (7)	174
N1—H1*B*⋯Br4^i^	0.89	2.79	3.442 (5)	132
N1—H1*B*⋯Br2^iii^	0.89	2.84	3.535 (5)	136
N1—H1*C*⋯Br3^i^	0.89	2.63	3.436 (5)	152
N4—H4⋯Br1^iv^	0.86	2.73	3.419 (5)	138

Symmetry codes: (i) 
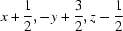
; (ii) 
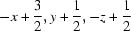
; (iii) 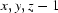; (iv) 
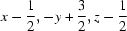
.

**Fig. 1.**
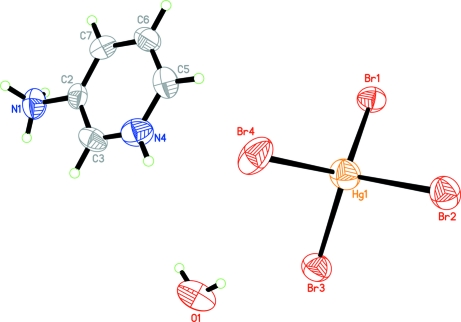
A view of the asymmetric unit with the atom-numbering scheme. Displacement ellipsoids are drawn at the 50% probability level.

**Fig. 2.**
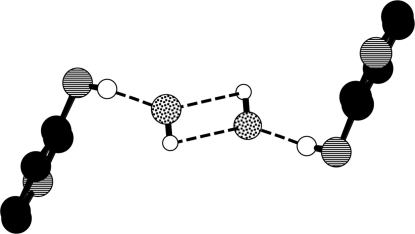
A cation···2H2O···cation supramolecular motif, via significant N—H···O and O—H···O hydrogen bonding. Hydrogen bonds are shown as dashed lines.

**Fig. 3.**
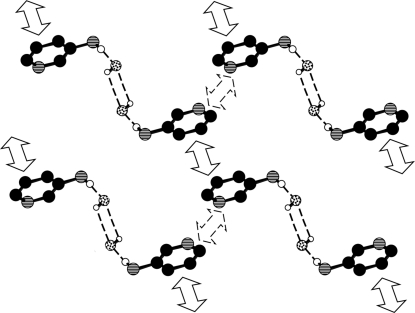
Cationic sheets in the ac plane. π···π stacking is represented as double headed arrows. Solid double headed arrows are intra-layer interactions, while dashed double headed arrows are inter-layers interactions.

**Fig. 4.**
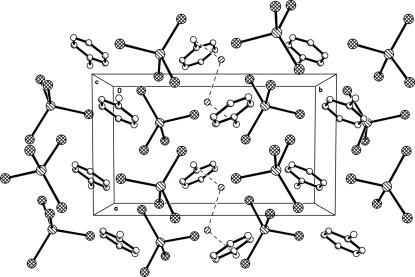
Overall packing of alternating anion stacks and sheets of cations and water molecules. Hydrogen atoms omitted for clarity.

## supplementary crystallographic information

### Comment

Noncovalent interactions play an important role in organizing structural units in both natural and artificial systems (Desiraju, 1997). In connection with ongoing studies (Al-Far *et al.*, 2006; Al-Far & Ali 2007*a*,*b*; Ali & Al-Far 2007*a*,*b*; Ali & Al-Far 2008; Ali *et al.*, 2008) of the structural aspects of bromometal anions' salts, we herein report the crystal structure of the title compound.

In the title compound, Fig. 1, the asymmetric unit contains one cation and one anion along with one water molecules. The anion exhibits a distorted tetrahedral arrangement about Hg atom (Table 1). The Hg—Br1 and Hg—Br4 [2.5875 (6) and 2.5818 (7) Å, respectively] bonds are almost invariant and significantly shorter than Hg—Br2 and Hg—Br3 [2.6309 (7) and 2.6216 (7) Å, respectively]. These lengths fall within the range of Hg—Br distances reported previously for compounds containing [HgBr_4_]^2-^ anions (Al-Far *et al.*, 2006; Ali & Al-Far 2008). It is noteworthy that the longer Hg—Br2, Br3 bonds are involved in more interactions than the shorter ones (Table 2). In the cation, the bond lengths and angles are in accordance with normal values (Orpen *et al.*, 1989). The cation is, of course, planar, in which N1 and N2 atoms are also coplanar.

The packing can be regarded as sheets of cations in the *ac* plane that are separated by stacks of anions.

Each two cations are connected *via* two water centers in a cation···2H_2_O···cation supramolecular motif, (Fig. 2), through N—H···O and O—H···O hydrogen bonding. These motifs are further connected to the next one *via*π···π stacking leading to infinite layers of ···pyNH_3_···OH_2_···H_2_O···H_3_Npy··· pyNH_3_···OH_2_···H_2_O···H_3_Npy··· connected molecules (Fig. 3). These layers are then connected by π···π stacking to the next layer causing the sheet arrangement (Fig. 3). The sheets are separated by the anion stacks (Fig. 4), where no significant Br···Br interactions (shortest Br···Br is 3.9527 (9) Å) were observed. The anion stacks are interacting extensively with cation sheets by different significant hydrogen bonds of the type pyN—H···Br and C—H···Br (Table 2), along with extra O—H···Br—Hg interactions (Table 2), cause to the formation of a three-dimensional supramolecular architecture. π···π Stacking may be effective in the stabilization of the crystal structure apart from hydrogen bonding, dipole-dipole and van der Waals interactions.

### Experimental

A warm solution of HgCl_2_ (1.0 mmol) dissolved in ethanol (10 ml) and HBr (60%, 3 ml), was added dropwise to a stirred hot solution of 2-aminopyridine (1 mmol) dissolved in ethanol (10 ml). After refluxing for 2 h, the mixture was filtered off, and then allowed to stand undisturbed at room temperature. The salt crystallized over 3 days as pink crystals. Crystals were filtered off and one crystal suitable for diffraction measurements was used to collect data.

### Refinement

H atoms attached to water O atoms were located in a difference map and refined with restraints (O—H distance of 0.89 Å). Other H atoms were positioned geometrically, with N—H = 0.86 Å (for py NH), N—H = 0.89 Å (for ammonium NH) and C—H = 0.93 Å for aromatic H, and constrained to ride on their parent atoms, with *U*_iso_(H) = 1.2*U*_eq_(C,*N*).

### Crystal data

**Table:**

(C_5_H_8_N_2_)[HgBr_4_]·H_2_O	*F*_000_ = 1128
*M**_r_* = 634.34	*D*_x_ = 3.181 Mg m^−^^3^
Monoclinic, *P*2_1_/*n*	Mo *K*α radiation λ = 0.71073 Å
Hall symbol: -P 2yn	Cell parameters from 5049 reflections
*a* = 8.1896 (7) Å	θ = 2.3–27.6º
*b* = 14.0245 (12) Å	µ = 23.66 mm^−^^1^
*c* = 11.5711 (10) Å	*T* = 296 (2) K
β = 94.730 (2)º	Chunk, pink
*V* = 1324.5 (2) Å^3^	0.20 × 0.10 × 0.03 mm
*Z* = 4	

### Data collection

**Table:**

Bruker–Siemens SMART APEX diffractometer	3780 independent reflections
Radiation source: fine-focus sealed tube	2628 reflections with *I* > 2σ(*I*)
Monochromator: graphite	*R*_int_ = 0.056
Detector resolution: 8.3 pixels mm^-1^	θ_max_ = 30.1º
*T* = 296(2) K	θ_min_ = 2.3º
ω scans	*h* = −11→11
Absorption correction: multi-scan(SADABS; Bruker, 2001)	*k* = −19→19
*T*_min_ = 0.034, *T*_max_ = 0.492	*l* = −16→16
16873 measured reflections	

### Refinement

**Table:**

Refinement on *F*^2^	Secondary atom site location: difference Fourier map
Least-squares matrix: full	Hydrogen site location: inferred from neighbouring sites
*R*[*F*^2^ > 2σ(*F*^2^)] = 0.035	H atoms treated by a mixture of independent and constrained refinement
*wR*(*F*^2^) = 0.074	*w* = 1/[σ^2^(*F*_o_^2^) + (0.018*P*)^2^ + 0.74*P*] where *P* = (*F*_o_^2^ + 2*F*_c_^2^)/3
*S* = 1.03	(Δ/σ)_max_ = 0.001
3780 reflections	Δρ_max_ = 0.85 e Å^−^^3^
126 parameters	Δρ_min_ = −1.32 e Å^−^^3^
3 restraints	Extinction correction: SHELXTL (Sheldrick, 2008), Fc^*^=kFc[1+0.001xFc^2^λ^3^/sin(2θ)]^-1/4^
Primary atom site location: structure-invariant direct methods	Extinction coefficient: 0.00271 (13)

### Fractional atomic coordinates and isotropic or equivalent isotropic displacement parameters (Å^2^)

**Table:**

	*x*	*y*	*z*	*U*_iso_*/*U*_eq_	
O1	0.8331 (6)	0.5315 (4)	0.4741 (5)	0.0770 (15)	
H1	0.883 (9)	0.575 (4)	0.433 (5)	0.116*	
H2	0.802 (10)	0.560 (4)	0.538 (4)	0.116*	
Hg1	0.68459 (3)	0.763019 (18)	0.84039 (2)	0.05085 (10)	
Br1	0.95522 (7)	0.85483 (4)	0.89061 (5)	0.04424 (15)	
Br2	0.53644 (7)	0.74688 (4)	1.03273 (5)	0.04900 (16)	
Br3	0.74834 (8)	0.59454 (4)	0.75688 (5)	0.05157 (17)	
Br4	0.45943 (8)	0.83129 (5)	0.69416 (6)	0.0622 (2)	
N1	0.8418 (6)	0.9030 (4)	0.1560 (4)	0.0506 (13)	
H1A	0.7884	0.9447	0.1089	0.076*	
H1B	0.8181	0.8440	0.1317	0.076*	
H1C	0.9492	0.9128	0.1560	0.076*	
C2	0.7926 (6)	0.9151 (4)	0.2726 (4)	0.0337 (11)	
C3	0.6981 (6)	0.8466 (4)	0.3172 (5)	0.0413 (13)	
H3	0.6637	0.7934	0.2737	0.050*	
N4	0.6563 (6)	0.8582 (4)	0.4258 (4)	0.0501 (12)	
H4	0.5957	0.8158	0.4547	0.060*	
C5	0.7057 (7)	0.9339 (4)	0.4914 (5)	0.0462 (14)	
H5	0.6764	0.9390	0.5672	0.055*	
C6	0.7989 (7)	1.0030 (4)	0.4463 (5)	0.0424 (13)	
H6	0.8318	1.0562	0.4903	0.051*	
C7	0.8433 (7)	0.9933 (4)	0.3356 (5)	0.0419 (13)	
H7	0.9074	1.0395	0.3036	0.050*	

### Atomic displacement parameters (Å^2^)

**Table:**

	*U*^11^	*U*^22^	*U*^33^	*U*^12^	*U*^13^	*U*^23^
O1	0.061 (3)	0.073 (4)	0.096 (4)	−0.012 (3)	0.003 (3)	−0.027 (3)
Hg1	0.04444 (15)	0.05348 (17)	0.05300 (16)	−0.00161 (11)	−0.00571 (10)	−0.00193 (11)
Br1	0.0406 (3)	0.0417 (3)	0.0508 (3)	−0.0052 (2)	0.0063 (2)	−0.0018 (2)
Br2	0.0451 (3)	0.0563 (4)	0.0451 (3)	−0.0050 (3)	0.0014 (3)	−0.0036 (3)
Br3	0.0573 (4)	0.0473 (4)	0.0506 (3)	−0.0023 (3)	0.0077 (3)	−0.0071 (3)
Br4	0.0454 (3)	0.0673 (5)	0.0713 (4)	−0.0031 (3)	−0.0099 (3)	0.0294 (3)
N1	0.053 (3)	0.061 (3)	0.037 (3)	0.010 (2)	0.003 (2)	0.000 (2)
C2	0.034 (3)	0.039 (3)	0.027 (2)	0.008 (2)	−0.001 (2)	0.002 (2)
C3	0.030 (3)	0.040 (3)	0.053 (3)	−0.004 (2)	−0.003 (2)	−0.012 (3)
N4	0.041 (3)	0.048 (3)	0.062 (3)	−0.009 (2)	0.007 (2)	0.003 (2)
C5	0.045 (3)	0.057 (4)	0.036 (3)	0.001 (3)	0.003 (2)	−0.004 (3)
C6	0.045 (3)	0.038 (3)	0.044 (3)	−0.003 (2)	−0.001 (3)	−0.009 (2)
C7	0.045 (3)	0.034 (3)	0.047 (3)	0.000 (2)	0.006 (3)	0.006 (2)

### Geometric parameters (Å, °)

**Table:**

O1—H1	0.89 (6)	C2—C3	1.362 (7)
O1—H2	0.89 (6)	C3—N4	1.339 (7)
Hg1—Br4	2.5818 (7)	C3—H3	0.9300
Hg1—Br1	2.5875 (6)	N4—C5	1.349 (7)
Hg1—Br3	2.6216 (7)	N4—H4	0.8600
Hg1—Br2	2.6309 (7)	C5—C6	1.364 (8)
N1—C2	1.450 (6)	C5—H5	0.9300
N1—H1A	0.8900	C6—C7	1.367 (7)
N1—H1B	0.8900	C6—H6	0.9300
N1—H1C	0.8900	C7—H7	0.9300
C2—C7	1.363 (7)		
			
H1—O1—H2	108 (5)	N4—C3—C2	117.8 (5)
Br4—Hg1—Br1	120.99 (2)	N4—C3—H3	121.1
Br4—Hg1—Br3	104.23 (2)	C2—C3—H3	121.1
Br1—Hg1—Br3	109.78 (2)	C3—N4—C5	122.4 (5)
Br4—Hg1—Br2	103.42 (2)	C3—N4—H4	118.8
Br1—Hg1—Br2	107.40 (2)	C5—N4—H4	118.8
Br3—Hg1—Br2	110.73 (2)	N4—C5—C6	119.7 (5)
C2—N1—H1A	109.5	N4—C5—H5	120.2
C2—N1—H1B	109.5	C6—C5—H5	120.2
H1A—N1—H1B	109.5	C5—C6—C7	119.3 (5)
C2—N1—H1C	109.5	C5—C6—H6	120.4
H1A—N1—H1C	109.5	C7—C6—H6	120.4
H1B—N1—H1C	109.5	C2—C7—C6	119.2 (5)
C7—C2—C3	121.6 (5)	C2—C7—H7	120.4
C7—C2—N1	119.7 (5)	C6—C7—H7	120.4
C3—C2—N1	118.7 (5)		
			
C7—C2—C3—N4	−0.2 (8)	N4—C5—C6—C7	−1.3 (9)
N1—C2—C3—N4	178.8 (5)	C3—C2—C7—C6	0.3 (8)
C2—C3—N4—C5	−0.7 (8)	N1—C2—C7—C6	−178.7 (5)
C3—N4—C5—C6	1.4 (9)	C5—C6—C7—C2	0.4 (8)

### Hydrogen-bond geometry (Å, °)

**Table:**

*D*—H···*A*	*D*—H	H···*A*	*D*···*A*	*D*—H···*A*
O1—H1···Br2^i^	0.89 (6)	2.98 (6)	3.564 (5)	125 (5)
O1—H2···Br3	0.89 (6)	2.65 (3)	3.513 (5)	162 (7)
N1—H1A···O1^ii^	0.89	1.80	2.686 (7)	174
N1—H1B···Br4^i^	0.89	2.79	3.442 (5)	132
N1—H1B···Br2^iii^	0.89	2.84	3.535 (5)	136
N1—H1C···Br3^i^	0.89	2.63	3.436 (5)	152
N4—H4···Br1^iv^	0.86	2.73	3.419 (5)	138

Symmetry codes: (i) *x*+1/2, −*y*+3/2, *z*−1/2; (ii) −*x*+3/2, *y*+1/2, −*z*+1/2; (iii) *x*, *y*, *z*−1; (iv) *x*−1/2, −*y*+3/2, *z*−1/2.
